# Allele-specific expression and eQTL analysis in mouse adipose tissue

**DOI:** 10.1186/1471-2164-15-471

**Published:** 2014-06-13

**Authors:** Yehudit Hasin-Brumshtein, Farhad Hormozdiari, Lisa Martin, Atila van Nas, Eleazar Eskin, Aldons J Lusis, Thomas A Drake

**Affiliations:** Department of Medicine/Division of Cardiology, David Geffen School of Medicine, University of California, Los Angeles, CA 90095 USA; Department of Computer Science, University of California, Los Angeles, CA 90095 USA; Department of Pathology and Laboratory Medicine, University of California, Los Angeles, CA 90095 USA

**Keywords:** *Cis*, *Trans*, eQTL, Allele Specific Expression, Adipose, RNA-seq, DNase I hypersensitivity, DBA/2J, C57BL/6J

## Abstract

**Background:**

The simplest definition of *cis*-eQTLs versus *trans,* refers to genetic variants that affect expression in an allele specific manner, with implications on underlying mechanism. Yet, due to technical limitations of expression microarrays, the vast majority of eQTL studies performed in the last decade used a genomic distance based definition as a surrogate for *cis,* therefore exploring *local* rather than *cis-*eQTLs*.*

**Results:**

In this study we use RNAseq to explore allele specific expression (ASE) in adipose tissue of male and female F1 mice, produced from reciprocal crosses of C57BL/6J and DBA/2J strains. Comparison of the identified *cis*-eQTLs, to *local*-eQTLs, that were obtained from adipose tissue expression in two previous population based studies in our laboratory, yields poor overlap between the two mapping approaches, while both *local*-eQTL studies show highly concordant results. Specifically, *local*-eQTL studies show ~60% overlap between themselves, while only 15-20% of *local*-eQTLs are identified as *cis* by ASE, and less than 50% of ASE genes are recovered in *local*-eQTL studies. Utilizing recently published ENCODE data, we also find that ASE genes show significant bias for SNPs prevalence in DNase I hypersensitive sites that is ASE direction specific.

**Conclusions:**

We suggest a new approach to analysis of allele specific expression that is more sensitive and accurate than the commonly used fisher or chi-square statistics. Our analysis indicates that technical differences between the *cis* and *local*-eQTL approaches, such as differences in genomic background or sex specificity, account for relatively small fraction of the discrepancy. Therefore, we suggest that the differences between two eQTL mapping approaches may facilitate sorting of SNP-eQTL interactions into true *cis* and *trans,* and that a considerable portion of *local*-eQTL may actually represent *trans* interactions.

**Electronic supplementary material:**

The online version of this article (doi:10.1186/1471-2164-15-471) contains supplementary material, which is available to authorized users.

## Background

Allele specific expression (ASE) refers to unequal expression of multiple alleles of a gene in a given organism. The extreme case of ASE is monoallelic expression, where only one of the alleles is expressed while the other is completely inactive. While known examples of monoallelic expression usually reflect active epigenetic regulation, such as chromosome X inactivation, olfactory receptor silencing or parental imprinting, less pronounced ASE encompasses a continuous spectrum that seems to arise due to genetic variation in regulatory regions.

Effects of genetic variation on gene expression has been the subject of numerous studies in humans [1–3], mice [4, 5] and other organisms. Collectively, loci in which expression level is influenced by specific genetic variation are termed expression quantitative trait loci (eQTL), and these associations are commonly divided into *cis*-eQTLs and *trans*-eQTLs. In their classical definition *cis*-eQTLs refers to genetic variants that affect expression of a locus on the same DNA molecule, thus in allele-specific fashion, while *trans*-eQTLs are expected to affect gene expression in an allele independent manner [6]. These terms imply mechanistic differences between the two classes of eQTLs, in terms how the effect of genetic variation is translated into expression differences.

Development of rapid and inexpensive high throughput hybridization based techniques for genome wide expression and genotyping facilitated genome-wide identification of eQTLs in multiple organisms. In most of these studies genome-wide transcript levels were measured by microarrays across a population, which was also genotyped for the available genetic markers [7, 8]. These types of linkage or association studies have identified eQTLs for hundreds to thousands of expressed loci in a given tissue, and showed that variation proximal to the gene often has greater effect and stronger association than more distal variations [9, 10]. Importantly, although microarrays generally do not measure gene expression in an allele-specific manner, a commonly accepted assumption in eQTL studies is that *local* associations likely act in a *cis* fashion; therefore, *local* and *distal* associations are often termed *cis* and *trans* respectively in the literature [11].

A second advance in genome wide expression profiling was recently achieved by RNAseq. In this technique (reviewed in [12]) RNA expression levels are measured by direct sequencing of RNA in the sample, and quantitative expression levels are derived from number of reads mapping to each locus. RNAseq and microarrays yield comparable results in terms of overall gene expression levels, and the comparison of the two techniques has been the subject of several studies [13]. Yet, in the realm of eQTL mapping RNAseq offers one significant advantage - it allows not only quantification of gene expression, but it also allows quantification of allele specific expression (ASE), if the expressed sequences of the two alleles differ by at least one base, by directly counting the reads with each one of the alleles in heterozygous samples. Therefore, RNAseq allows the distinction of true *cis*-eQTLs from *local* effects acting in *trans*.

To date, several studies that analyzed genome wide ASE in human, mice and cell lines [14–19] identifying hundreds to few thousands of genes that show significant imbalance in expression of the two alleles. Overall, this number of genes with significant *cis-*eQTLs is comparable to numbers identified by *local*-eQTLs. However the few studies that mention a comparison between *cis* and *local* eQTLs suggest that the overlap is low, i.e. 30-50%, with the difference typically attributed to technical differences, analytic limitations and potential immaturity of RNAseq based methods. Indeed, while quantification of ASE via RNA-seq is potentially very powerful and accurate [19], it is still a new and developing technique for which the appropriate statistical approaches are debated [14, 15, 20].

**Figure 1:**
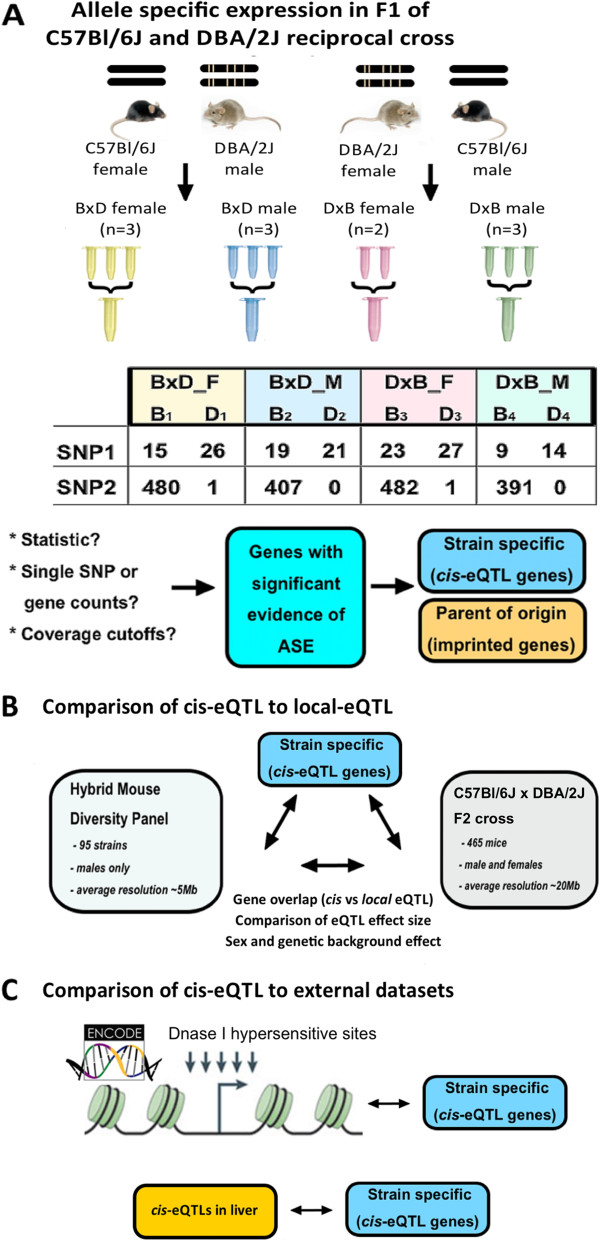
**Study summary.** This study explores methodological aspects related to interpretation of allele specific expression (ASE) from RNAseq data, as well as a broader question of *local* and *cis*-eQTL matching and their correspondence to independent set of identified DNase I peaks. **A**. Experimental approach to allele specific expression. Four pooled F1 samples from males and females of BxD and DxB crosses were sequenced. Allele specific counts for each SNP in each sample were produced in a table form, and treated as independent replicates in analysis by DEseq package. **B**. Summary of previous *local*-eQTL studies and the performed comparisons. Hybrid Mouse Diversity Panel is a panel of 95 inbred and recombinant inbred strains, that was previously used for mapping *local*-eQTLs at ~5 Mb resolution. F2 cross refers to 465 male and female mice that were produced from B and D strains, and allowed a lower resolution *local*-eQTL mapping. **C**. In the final part of this study we compared *cis* and *local*-eQTLs to DNase I peaks, which potentially mark *cis* acting elements and to previously published ASE from another tissue. DNase I peak illustration was adapted form [21].

In this study, we explored *cis*-eQTLs and *local*-eQTLs in adipose tissue of DBA/2J, C57BL/6J (hereafter referred as D and B respectively) and their F1 reciprocal cross mice (DxB and BxD). First we used ASE in 4 samples - male and female DxB and BxD F1 mice to identify imprinted and *cis* affected genes. We then used the results of two large independent and complimentary *local*-eQTL mapping studies previously performed in our laboratory to compare them to *cis*-eQTLs, as identified from the F1 mice. The general structure of our analysis is depicted in Figure 1.

## Results and discussion

### Allele specific expression in F1 mice

The first part of this study focused on deriving a list of genes that show evidence of ASE in gonadal adipose tissue of F1 mice (Figure 1A). To this end we sequenced 4 RNA samples of F1 males and females from reciprocal cross of B and D mice (each sample was an equimolar pool of total RNA from 3 animals, with one exception which contained only 2 animals). Every sample was sequenced on a single lane of Illumina GAII instrument, using a 50 bp paired-end sequencing module, resulting in 90–100 million pairs of reads per sample. Reads were mapped against the mouse mm9 genome, allowing up to 3 mismatches per read. Sequencing counts and mapping parameters are summarized in Table 1.

**Table 1:** **Summary of mapping of RNAseq data used to analyze allele specific expression in F1 cross of B and D**

Cross	B × D	D × B	B × D	D × B
Sex	Female	Female	Male	Male
Animals per pool	3	2	3	3
Total reads	233,502,200	207,580,666	249,032,306	169,565,576
Reads passing QC	194,608,754	176,900,074	202,559,286	149,205,688
(%)	(83.34%)	(85.22%)	(81.34%)	(87.99%)
Mapped reads	153,220,639	138,009,910	144,153,858	107,732,312
(%)	(78.73%)	(78.02%)	(71.17%)	(72.2%)
Reads mapping to known SNPs (%)	4,761,915	4,598,505	4,617,955	3,733,381
Reads mapping to exonic SNPs (%)*	(85.5%)	(87.64%)	(87.08%)	(88.48%)
B to D mapping ratio (independent SNPs)	1.17 (1.09)	1.21 (1.15)	1.15 (1.08)	1.16 (1.08)
Covered SNPs	201,718	180,513	179,535	153,854
Covered Genes	9,877	9,679	9,796	9,492

*B and D refer to C57BL/6J and DBA/2J strains that differ at 1,834,754 SNPs in 17,002 genes. However, vast majority of these SNPs (>95%) are annotated as intronic, and therefore not necessarily expected to be covered by RNAseq. For simplicity sake, we call all 80,652 non-intronic SNPs “exonic”, while some of them fall within the untranslated regions and are not exonic per se.

Quantifying ASE from sequencing data is a promising emerging technique which has not yet matured to have an accepted standard analysis pipeline. Variation in analysis mainly focuses on three aspects: (1) filtering of close and low coverage SNPs, (2) choice of statistical test and (3) choice between SNP specific counts or aggregate of allele specific counts across the haplotype. We explored the potential effect of all of those aspects on ASE calling in our data (these technical analyses are fully described in Additional file 1: Materials and Methods), and concluded that aggregate counts over gene regions of exon SNPs, analyzed with RNAseq expression package DEseq, provides both better sensitivity and accuracy for ASE than using the sample specific Fisher exact test and overlapping of sample specific results. One of the major problems in assessment of ASE is estimation of error. We used the inherent biological structure of gene transcription to assess the accuracy of our method - i.e. at the SNP level we looked at pairs of SNPs within the same exon that show significant and discordant allele specific expression. Similarly, when aggregating counts we looked at pairs of exons within the same gene that show significant allele specific expression with opposite direction of imbalance. Altogether we conclude that aggregating allele specific SNP counts over a haplotype produces more robust and accurate measure of ASE, than SNP by SNP based approaches.

Our method of ASE detection implements the DEseq package [22], specifically developed for RNAseq differential expression analysis, to variant specific counts. The analysis accounts for global differences in library coverage and count distribution, and uses estimation of variance between biological replicates of the same condition to assign a p-value for differential expression between the tested conditions. When applied to allele specific counts on a gene, rather than SNP by SNP level, this approach effectively tests allelic imbalance of gene expression.

Inherent to this method, adding biological replicates results in more accurate variance estimation, and therefore in more sensitive differential expression statistics, typically reflected in higher proportion of differentially expressed genes when having more samples. This dependency is expected to even out when the number of replicates is high, i.e. when additional replicates contribute very little to the variance estimation. Indeed, we looked into the effect of number of replicates on differential ASE calling and could clearly see that the increase in number of ASE genes was directly proportional to the increase in number of biological replicates analyzed. Using 4 replicates in such analyses resulted in higher sensitivity than Fisher exact test approach, and lower false positive rate (overall identifying ~1.5× more genes with allele specific expression, Additional file 1: Figure S4).

**Figure 2:**
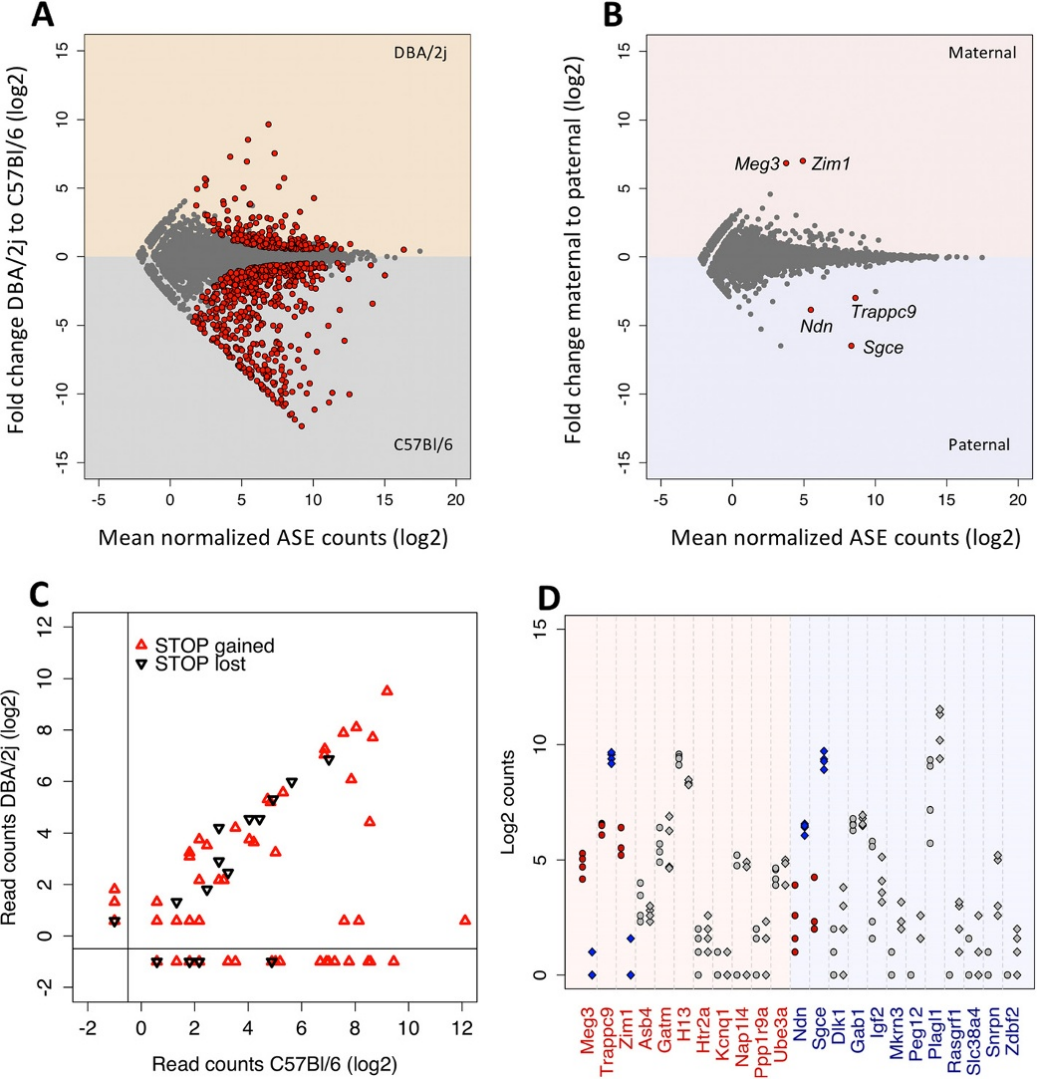
**ASE in F1. A** and **B** depict allelic imbalance (expressed as log2 fold change) versus mean expression, using either parent of origin **(A)** or strain **(B)** as the main variable. Genes with significant allelic imbalance (FDR adjusted p-value < 0.05) are in red. Significant *cis*-eQTLs were identified for 1085 genes **(A)** while parental imbalance was significant only for 5 genes **(B)**, plots backgrounds are shaded and labeled according to the direction of allelic imbalance. **C**. Allelic expression of variants resulting in premature stop codons (n = 60, red), or in loss of stop codons (n = 16, black). **D**. Allelic expression of all known imprinted genes detected in our samples. Gene name color and plot background indicated the expected expressed allele (red = maternal, blue = paternal). Dots and diamonds indicate expression of the maternal and paternal allele, respectively. Red and blue filled shapes indicate genes that show significant allelic imbalance (in red in B), grey filled shapes indicate that the observed difference does not reach 0.05 significance level.

We examined allele specific expression of 7988 genes (Additional file 2), and identified 1085 genes where either B or D alleles were significantly overexpressed (at FDR adjusted p-value cutoff of 0.05). These represent *cis-*eQTL genes in the classic definition of this term. In comparison, only 5 genes showed significant parental imbalance (Figure 2B, 2D and S6C), all of which were previously reported as imprinted, and the expressed allele in our data is the expected direction for all but one gene – *Trappc9. Trappc9* is a maternally expressed activator of NF-kappa-B pathway, mutations in which are associated with mental retardation [23–25]. In our data it shows paternal expression, consistent with similar result previously observed in human cells [26]. Allele specific expression of other known imprinted genes generally either conferred with the expected direction, but did not reach significance or showed comparable expression of both alleles (Figure 2D).

Sex is an important factor in gene expression that influences overall expression of hundreds of genes [27, 28]. Thus, in addition to analyzing the 4 samples together we also looked at the sex specificity of ASE, by analyzing ASE in male or female samples separately. Sex-restricted analysis of *cis-*eQTLs identified 854 *cis*-eQTL genes in males and 660 in females, with 598 genes overlapping between the two (Additional file 1: Figure S6B), and the vast majority (94%) were recapitulated in the combined analysis. As expected, using both sexes for analysis yielded overall higher number of *cis*-eQTLs than any of the sex restricted comparisons, reflecting the increase in power which stems from adding biological replicates. Comparison of fold change of ASE between male and female samples (Additional file 1: Figure S6A) indicated that ASE ratios are highly concordant between the sexes (R^2^ = 0.93, p-value <2.2e-16). Only a small number of genes show sex specific ASE (21 in males and 5 in females) which is likely due to fluctuations in ASE ratios at the low expression levels. The most pronounced effect of sex on ASE was observed in *Apba2* (amyloid beta (A4) precursor protein-binding, family A, member 2) where the ratio of D to B expression in females is 29 while in males it is only 1.3 (Additional file 1: Figure S6C). Furthermore, consistent with the presented allele specific data, in a previously analyzed BxD cross a significant eQTL is observed for *Apba2* only in females, but not in males ( http://systems.genetics.ucla.edu/data/mouse). However, the *Apba2* locus had only few allele specific reads (average of 10 per sample), therefore the difference in ASE ratios of *Apba2* between males and females should be regarded with caution. In addition, analysis of sex specific imprinting showed male specific imprinting of genes that reside on chromosome X, reflecting the maternal heritage of the sex chromosome. However we did not identify any other genes imprinted in sex specific manner (Figure S6C).

A set of potentially functional variants are those that result in premature stop codons. Transcripts carrying such mutations are expected to be actively degraded through nonsense mediated decay (reviewed in [29]), however the generality of this mechanism is unknown. A recent paper on ASE in human cell lines, suggested that up to 68% of variants expected to result in nonsense mediated decay escape this fate via unidentified mechanisms [30]. Therefore we examined ASE of potential premature stop codons in our data. Since mouse reference genome correspondence to B strain is, in a way, a random assignment, we explored ASE of variants annotated both as stop-gain (n = 60) and stop-loss (n = 16) in D strain. Consistent with the recent data published from humans a majority of those variants show close to equal expression of both alleles (Figure 2C), with only ~30% showing a marked reduction in the expression of impaired allele.

### Comparison of ASE and *local*-eQTL mapping approaches (HMDP and F2)

Previous work in our lab yielded two datasets of *local*-eQTLs pertaining to adipose tissue in B and D strains. The first dataset is from the Hybrid Mouse Diversity Panel (HMDP), a resource of many inbred and recombinant inbred strains which was extensively described in [31]. Adipose expression data was collected from males of 95 inbred strains (28 classical and 67 recombinant inbred strains) recently published in Parks et al. [32]. In short, *local*-eQTLs in the HMDP were analyzed by association mapping between expression probes and SNPs within 2 Mb window of the probe, choosing the SNP with the best p-value within each window (details of the analysis are in [32]). Using this approaches we examined 12703 genes, identifying *local*-eQTL for 3744 (29%) of the genes, 1991 of which are associated with a SNP polymorphic in D. Since both F1 ASE and the F2 *local*-eQTL were derived from mice on B and D background, we limited HMDP analysis to genes that had an eQTL associated with a SNP polymorphic in D.

The second dataset was derived from genome wide expression of 21023 genes and genotyping 2637 SNPs in an F2 population derived from B and D strains, constructed on the background of a *db/db* mutation. This population includes 465 male and female mice for which liver and adipose expression data were collected. Extensive analysis of liver eQTLs of this cross is described in [7], where they estimated genome wide significance was estimated as LOD >6.2 using 1000 permutations. We subsequently adopted this threshold as significance level for the adipose eQTLs derived from the same mice. Similarly to liver data, adipose eQTL analysis identified 7622 genes with at least one genome wide significant eQTL, while most significant eQTLs (best association signal for each gene, Additional file 1: Figure S7A) showed an expected enrichment for the same chromosome and *local* (within 2 Mb) signals.

To make the datasets comparable, we then applied the same filters as for the HMDP *local*-eQTL identification to the F2, i.e. chose best local association for each gene. In this way we identified 3028 genes that had a significant *local*-eQTL in F2 data (Additional file 3). However, the resolution of the F2 cross is much lower than that of HMDP, because of extensive linkage disequilibrium which contributes to local effects. We therefore asked whether the *local*-eQTL was also the most significant genome-wide association for the gene. Indeed, 1588 genes with *local*-eQTL show a more significant association with another SNP on the same chromosome, and the effects of the local and more distal SNPs were almost identical (Rsq = 0.98, Additional file 1: Figure S7B), suggesting that they cannot be reliably distinguished. In contrast, only 111 out of 4118 genes for which the most significant eQTL is on a different chromosome also show a significant *local*-eQTL signal, and the effect of these signals does not seem to be correlated (Additional file 1: Figure S7B).

**Figure 3:**
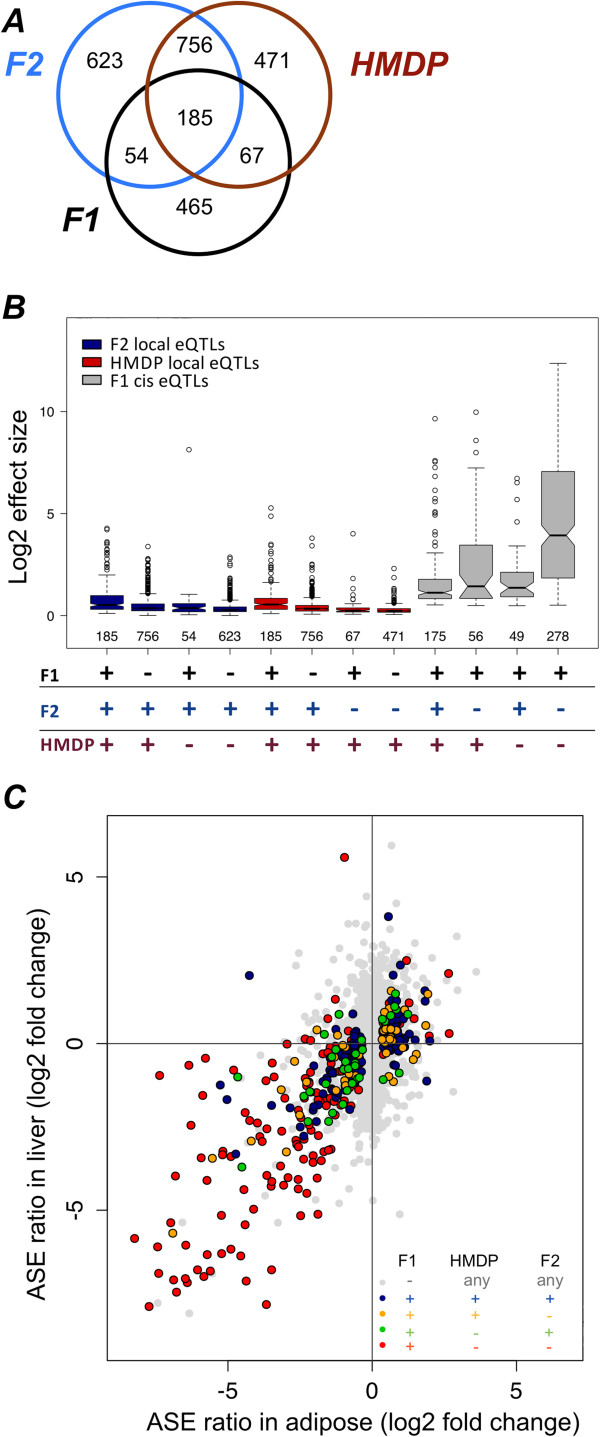
***Cis*** **versus** ***local*** **-eQTLs.** Comparison of *local* and *cis*-eQTL suggest a much better concordance between the two *local*-eQTL datasets, than of any of them to *cis*-eQTLs identified by ASE. In addition reproducibility of cis-eQTLs seems to be independent of their effect size (defined as ratio of expression between the two homozygous alleles). **A**. Overlap of *cis*-eQTLs identified in F1 dataset, with *local*-eQTLs identified in HMDP and F2 studies. F1, HMDP and F2 labels represent the respective datasets. **B**. Recovery of *local*-eQTLs, but not *cis*-eQTLs correlates to effect size of eQTL. Boxplot shows effect sizes of the different overlapping groups in different studies. Red indicates effect sizes in HMDP, blue in F2 and grey in F1. “+ ” indicates *local* or *cis*-eQTL, “-” indicates not significant. **C**. Correlation of ASE ratio in adipose and liver data. Genes are colored according to overlap with *local*-eQTL studies. Genes that do not show a significant *cis*-eQTL in adipose are in grey, other colors are as indicated in the figure - “+ ” indicates *local* or *cis*-eQTL, “-” indicates not significant.

Three way comparison of significant *local* and *cis*-eQTLs shows that the two *local*-eQTL mapping approaches were more concordant than either of them with *cis*-eQTL mapping by ASE (Figure 3A, p values for overlap between F1 and HMDP is p < 5.082e-10, for overlap between F1 and F2 is p < 5.436e-14 and for overlap between HMDP and F2 is p < 4.984e-262). Recovery rate of *cis*-eQTLs, by either of the --eQTL mapping approaches was less than 40%, while ASE was able to recover only 17% of *local*-eQTLs. In contrast, 58-63% of the *local*-eQTLs identified by one local mapping approaches are recovered in the other study. We also looked at the overlap of genes overexpressing specifically the D allele, since these are less likely to represent false positives in our study. In line with the overall results, *local*-eQTLs failed to reproduce 25-40% of these as well.

Notably the HMDP was derived from sex specific (male) expression data and on heterogeneous genetic background, while F2 and F1 are data derived from expression in both sexes, and limited to B and D genetic background. Nevertheless, the overlap between ASE and F2 was not better than the one with HMDP. To address this issue in a more explicit way, we examined the overlap of *local* and *cis*-eQTLs in pairwise comparisons of all the datasets (Additional file 1: Table S3), using either genome and sex matched or unmatched datasets. Restricting genomic background of *local*-eQTL from HMDP to those associated with SNP polymorphic between B and D strains reduced the proportion of *cis*-eQTLs recovered by HMDP, while restricting *cis*-eQTL analysis to male samples had virtually no effect on the results.

We then compared the effect sizes, defined as average fold change between two homozygous genotypes in F2 and HMDP, or the average fold change of expression between alleles in ASE data, between the different datasets. Effect sizes of *local*-eQTLs in F2 and HMDP yielded highly concordant results, while effect sizes of ASE have poorer correlation to effect sizes observed in *local*-eQTLs (Additional file 1: Figure S8A-C). As expected, *local*-eQTLs recovered by multiple approaches had higher effect sizes as compared to study-specific ones. Interestingly, *cis*-eQTLs not recovered by other approaches had higher effect sizes and were more variable than those recovered by *local*-eQTLs (Figure 3B and Additional file 1: Figure S8D). Notably, we did not observe any difference in total expression between *cis-*eQTLs recovered by *local*-eQTLs and those that are not (Additional file 1: Figure S8E). Together these results suggest that while F1 may lack power to discover the weaker local eQTL, the poor recovery rate of *cis*-eQTL by *local-*eQTLs is not due to lack of power of HMDP or F2 samples. To address this directly we looked whether increasing p-value cutoffs will improve the overlap between datasets. If the case is lack of power we expect that increasing the stringency of p-value cutoff, will increase the proportion of overlapping genes, and indeed this is the evident when comparing 2 *local*-eQTL studies (Additional file 1: Figure S10). However, comparing either *local*-eQTL studies to *cis*-eQTL does not improve the overlap at all (Additional file 1: Figure S10).

We then sought to further validate the newly identified *cis*-eQTLs. First, we compared ASE in adipose to ASE in liver of the same mice, recently published by our group ( [33]). The results show strong concordance (R^2^ = 0.62, p < 2.2e-16, Figure 3C) between ASE ratios in both tissues, even though the liver data were sequenced more shallowly. We then examined the distribution of SNPs within the genes in each group. Out of 465 genes 179 showed exclusive expression of the B allele, with 135 of these genes having only 1 exonic SNP. We then sequenced DNA from D mice to verify a subset of those SNPs at the genomic level (Table 2) and we manually examined the alignment of the subset of SNPs showing exclusive expression. We could not identify any problem in the alignment of reads in those regions, but none of the SNPs exhibiting B only expression were verified by Sanger sequencing. Importantly, this group of genes did not contribute to the observable effect sizes of ASE (Figure 3B), since only genes with expression of both B and D alleles were included in the boxplot. However, this raises a concern that genomic sequencing errors may cause false positive in the ASE data, even in genes that harbor multiple exonic SNPs. To address that, we removed all SNPs that showed either B or D exclusive expression from our data, and repeated our analysis at the gene level. This reduced the number of genes we could examine from 7988 to 6555, of which 624 showed significant ASE (Additional file 1: Table S4). However, intersecting those genes with the *local*-eQTL studies did not have a great impact on the overlap – i.e. only 50% of these *cis*-eQTLs also had a significant *local*-eQTL. Altogether this suggests that sequencing errors may have a substantial impact on ASE results, but are not the major source of discrepancy between *local* and *cis*-eQTL studies.

**Table 2:** **Sequencing validation of SNPs showing exclusive expression of B or D alleles**

Gene	chr	SNP base (mm9)	SNP type	Num reads	RNA seq allele	B (mm9)	D	D by sequence	Control/test	Conclusion
*Kcnk3*	5	30925772	3’_UTR	58	D	T	C	C	Control	TRUE
*1190005I06Rik*	8	123132603	3’_UTR	108	D	C	G	G	Control	TRUE
*Adra2c*	5	35622711	SYN	5	B	T	G	T	Test	FALSE
*Kcnd1*	X	7408739	NS	5.5	B	T	G	T	Test	FALSE
*Bcl6*	16	23974966	NS	53.25	B	T	C	T	Test	FALSE
*Rab4b*	7	27963863	5’_UTR	55.75	B	A	C	A	Test	FALSE
*Gnaq*	19	16207517	5’_UTR	58.25	B	A	G	A	Test	FALSE
*Psmd5*	2	34726327	NS	60.5	B	T	C	T	Test	FALSE
*Stat3*	11	100765040	SYN	160.75	B	G	A	G	Test	FALSE
*Fam100a*	16	4875477	3’_UTR	171.25	B	C	A	C	Test	FALSE
*Sub1*	15	11920770	STOP	173.75	B	T	A	T	Test	FALSE
*Atox1*	11	55263977	3’_UTR	352.25	B	T	A	T	Test	FALSE

### Comparison of *cis*and *local*-eQTL to DNase I hypersensitivity peaks

**Figure 4:**
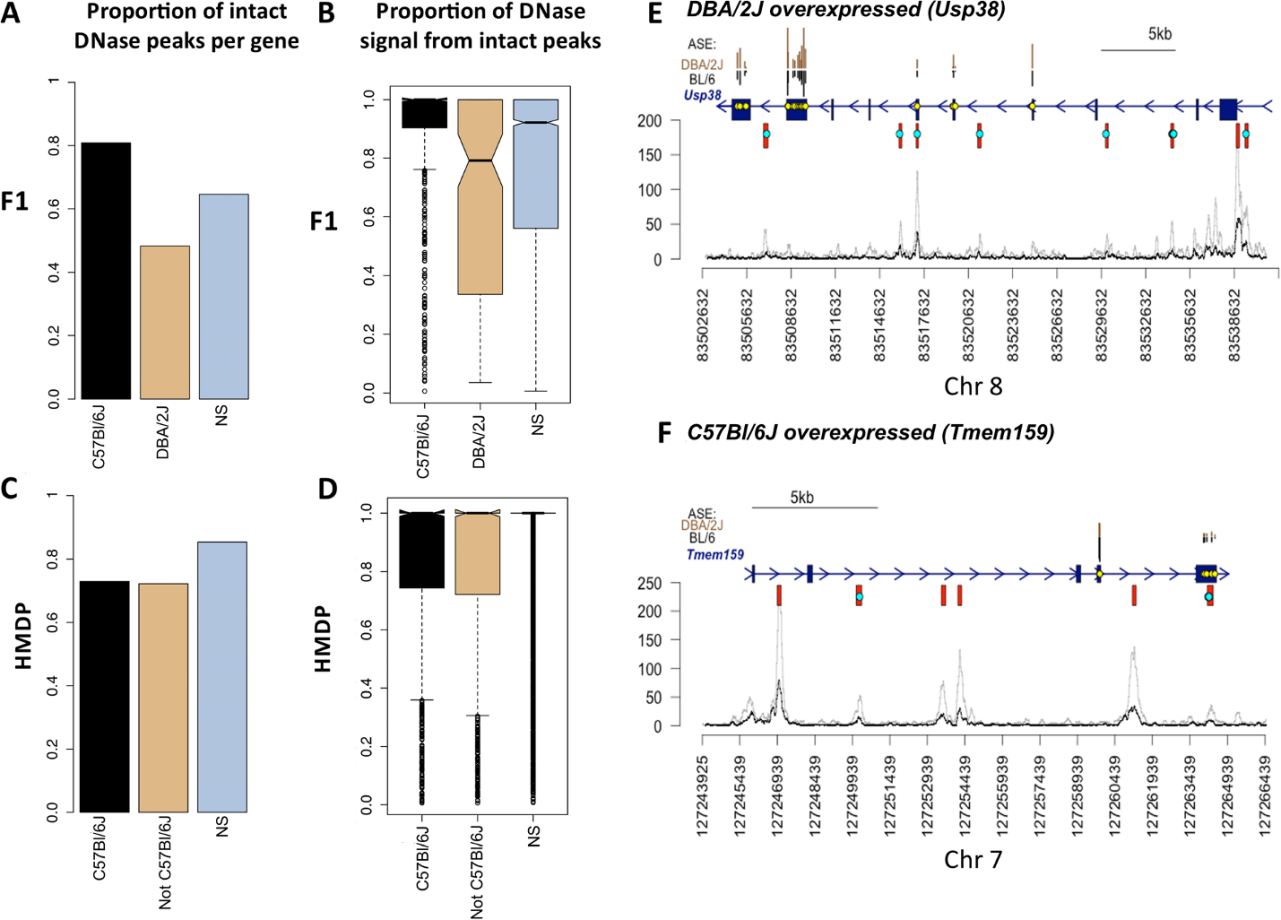
***Cis*** **, but not** ***local*** **-eQTLs correlate to polymorphisms in regions hypersensitive to DNase I.** ENCODE DNase I hypersensitivity data for mouse gonadal fat was obtained from UCSC genome browser. Conserved peaks were defined as overlapping peaks in the two replicates, and only these were used for the analysis. Genes were divided into three groups – eQTLs overexpressing the B allele (labeled B), eQTLs overexpressing the D allele in F1 study or the non B allele in the HMDP study, and genes that had no significant eQTL in the study (labeled NS). Intact peaks were defined as DNase I peaks that do not carry a polymorphism (SNP or indel) between **B** and **D** strains. For each gene we counted 1) total number of peaks 2) total number of intact peaks that overlap with the gene 3) sum of DNase I signal that comes from all peaks overlapping the gene and 4) sum of DNase I signal that comes only from intact peaks overlapping the gene. **A** and **C** show proportions of number of intact peaks per gene in F1 and HMDP data respectively. **B** and **D** show the average amount of DNase I signal obtained from those intact peaks. **E** and **F** illustrate *cis*-eQTLs that either overexpress the D allele (**E**, Usp38) or the B allele (**F**, Tmem159). Gene structure is shown in blue, conserved DNase I hypersensitive peaks are shown in red. Dots indicate SNPs that either overlap a conserved DNase I peak (cyan) or were used to produce allele specific counts (yellow). Original DNase I signal of replicates 1 and 2 is shown in black and grey, respectively. Allele specific counts for D and B are shown in brown and black on the top.

We then sought to relate our dataset to other datasets of *cis* acting elements. A common marker for *cis* acting elements are DNase I hypersensitive regions, which mark open chromatin. Multiple studies showed that these regions are associated with *cis* acting elements – such as promoters, transcription factor binding sites, insulators or enhancers, and genetic variation in those regions was shown to affect gene expression [34, 35]. Recently the ENCODE consortium published a plethora of data ( http://www.nature.com/encode), which explores the genomic landscape of these regions in human and mouse tissues as well as in cell lines. One of these datasets included DNase I hypersensitive peaks in gonadal fat tissue of two B male mice. Therefore, we predicted that genes with B or D overexpressed alleles will have a different proportion of *cis* acting elements carrying a SNP or indel in the D genome (Figure 4A). Indeed, when we compared the proportion of peaks carrying a SNP or when we quantified the relative amount of DNase I hypersensitivity signal coming from non-polymorphic sites in B or D overexpressed genes (Figure 4B), as measured in B mice, we see a significant difference between the two ASE groups. This effect was not observed for *local*-eQTLs identified in HMDP and analyzed in the same manner (Figure 4C,D). Figure 4 shows that while there is a clear difference between genes with *local-*eQTL and background (p-value < 2e-16), we see no particular difference between *local-*eQTLs based on the direction of overexpression, while we do observe strong difference in the *cis*-eQTL genes (p-value < 0.0001).

If we restrict the analysis to intersect of ASE genes that are also *local-*eQTLs, as expected we see that the DNase I signal in that group is less pronounced than in the *cis*-eQTL group, and more pronounced than in the *local-*eQTLs, being closer to the effect in *cis*-eQTLs. While we observe an effect in all groups of *cis-*eQTLs (based on overlap with *local*-eQTLs), the greatest effect is in the *cis-*eQTLs identified in F1 only.

## Conclusions

Following the era of genome-wide association studies, where the majority of variants associated with complex traits were found to be non-protein coding, and presumably regulatory, great effort is being invested in exploration of genetic variation that affects gene expression. In the recent years numerous publications have focused on the identification of “*cis*-eQTLs”, yet the vast majority of them define “*cis”* based on the distance between the genetic variant and the transcript, without direct evidence for ASE, thus in fact exploring *local* rather than *cis-*eQTLs. In this paper we carefully examined allele specific expression directly in adipose tissue of F1 heterozygous mice from parental B and D strains and compared it to previously identified *local*-eQTLs in two related mouse study cohorts. We identified >1000 genes that show evidence of *cis*-eQTL regulation in mouse adipose tissue of F1 mice, a number consistent with previous studies of ASE in various cell and tissue types. Hundreds of these were not identified by *local*-eQTL analyses in our F2 and HMDP cohorts, even though these approaches were sufficiently powered to identify the majority of such effects. Various limiting factors (e.g. different genetic background, sex matching, age matching) have been suggested as potentially significant contributors to the substantial discrepancy between *cis*-eQTL identified by ASE and *local*-eQTL mapping. However, our analysis suggests that the substantial difference between these may only in part be attributed to technical differences in sample ascertainment or analysis specifics. We observed limited effects of heterogeneous genetic background, as well as that of sex specificity, on concordance between *cis* and *local*-eQTL mapping, suggesting they are unlikely to be major contributing factors to this discrepancy.

Rather we propose that these are conceptually different and complimentary methods of identifying potential *cis*-regulation of gene expression. *Local*-eQTL mapping relies on variation in total gene expression between the three potential genotypes, while *cis*-eQTL mapping with ASE in heterozygotes relies on distribution of ratios of allele specific expression within each individual. The former compares differences in total expression assuming that other types of biological regulation and environmental factors acting in *trans*, will not mask or skew the differences in gene expression between the possible allelic combinations. Therefore, for *local*-eQTL approach to be successful, we need to assume that the strength of *local*-eQTL relative to all other regulators of gene expression is high, and that a relatively large number of individuals are studied. On the other hand, *cis*-eQTL mapping with ASE in heterozygotes reflects true *cis* effect in a completely matched, but specific environment of the heterozygous individual. It is theoretically possible to examine ASE in each and every individual separately. F1 mice represent a unique type of genomic situation where the animal is heterozygous for all variants that exist between the two strains. It is possible that some *cis* acting variants act in concert with *trans* acting variation, thus their effect would only be apparent if both are present. In such cases F1 provide the ideal genomic environment, since they are heterozygous for all possible *trans* and *cis* acting variants, while such effects would be more limited in F2 and HMDP mice. At the same time, in F1 mice both of the alleles share exactly the same genetic and cellular environment and ASE ideally represents true variation in transcription that arises from *cis* acting variation. Consequently *local*-eQTLs that are not replicated in ASE studies potentially represent *local trans* acting variants. We show evidence that effect size, and therefore sensitivity, has a significant impact on likelihood of *local*-eQTL being replicated by the *cis-*eQTL study. This is consistent with the generally accepted view that *cis*-eQTL have higher impact than *trans* acting variants on gene expression. Also, our approach to *cis*-eQTL mapping was limited by low number of samples, which restricts our conclusions. Nevertheless, we believe that the high proportion of non-replicated *local*-eQTLs, even when taking into account sex and genomic heterogeneity, together with the DNAse I peak data (discussed below) suggest that a considerable proportion of *local-*eQTLs may in fact act in *trans*.

Alternatively, the *local-*eQTLs studies were sufficiently powered to detect most *cis*-eQTL signals in the F1 data. Therefore, we suggest that *cis*-eQTLs, which are not replicated in *local-*eQTLs studies, represent genes where the relative contribution of *trans* acting factors to expression regulation is high, or instead where *cis* acting element resides far from the gene locus. The newly identified *cis-*eQTLs show higher effect, and larger variability of that effect, than the ones that replicate in *local*-eQTL studies, which is consistent with both explanations being relevant. The smaller effect sizes likely represent genes for which *trans* regulation plays a major role, while genes with strong *cis* acting elements suggest that some *cis* variation reside too far from the gene to be identified by *local*-eQTL mapping, consistent, for example with long range enhancer promoter interactions.

Finally, it is also possible that some of these genes represent false positive hits in our ASE analysis. Indeed, our sequencing results indicate that many of the genes with exclusive BL6 expression and only a single exonic SNP available for ASE analysis may reflect sequencing errors, resulting in somewhat inflated *cis*-eQTL numbers. However, we did not include genes with exclusive expression in the analysis of effect sizes, therefore this artifact is not a limiting factor of new *cis*-eQTLs with large effect sizes. In genes where multiple exonic SNPs are available, our analysis of discrepancies in ASE between exons within the same gene, and of SNPs within the same exon, indicates that false positive *cis*-eQTLs are not a prominent concern in our study.

Others previously suggested that *local*-eQTLs acting in *cis* map in general very close to transcription start site, and that variation in DNase I supersensitive sites accounts for much of the *cis* acting variants [35]. Interestingly, our ASE results correlate extremely well to genetic variation in independently acquired set of DNAse I sensitivity peaks in this tissue, which we believe provides an additional validation of the ASE approach. This signal indicates that DNAse I sites harboring a genetic variant between B and D strains, are associated with genes that show reduced expression of B allele in F1 mice. Additionally, we did not find a significant difference in the total number of DNase peaks or total DNase signals between the groups (data not shown) in B mice. This in general would be consistent with a situation where the total level of expression of a gene is driven by elements that are stronger than the effect of the *cis* acting variant, such as biological feedback loops. In ASE measurements the *cis* effect would still come to light, independently of the total expression (that may remain unaffected). Currently much of the research effort in biology focuses on generation of large, comprehensive genome wide datasets, such as ENCODE project. One of the aims of such efforts is being able to look for correlations between the biological signals, in hope that they will lead to better insight on how these signals translate into phenotypes. Classification of variants as *cis,* as opposed to *trans* has implication on the likely molecular mechanisms that are involved in its action. In this realm, identification of a comprehensive set of genes that are under true *cis* regulation is important when comparing different types of genome wide data associated with potential *cis*-active elements, such as DNase I peaks or histone markers, to actual gene expression. Misclassification of variants between the two groups will in turn lower the power of such studies, or lead to spurious results.

Nonsense mediated decay (NMD) is a well documented mechanism of transcript degradation in cases of premature stop codons or aberrant splice pattern (recently reviewed in [29]). Yet, only few studies have looked at the generality of this mechanism, and a recent human study suggested that most variants resulting in premature stop codons may escape NMD through unknown mechanisms [30]. Our data support the conclusion that only ~30% of such variants lead to significant reduction in expression of the impaired allele, suggesting that either NMD acts selectively only on specific transcripts or that there are additional, unknown mechanisms that allow transcripts to avoid it.

In addition to *cis*-eQTLs, ASE was effectively used to search for genes with parent of origin effects, commonly termed parental imprinting. Typically parental imprinting is spatially and temporally restricted, and outside of these restrictions imprinted genes tend to be expressed from both alleles. However, due to the monoallelic expression in certain tissues and developmental stages, mutations in one of the alleles may act as dominant and lead to devastating phenotypes [36–38]. Earlier studies had identified only ~150 mouse genes that show either maternal or paternal expression ( http://igc.otago.ac.nz/home.html, http://www.geneimprint.com/). Other studies suggested based on ASE that his may be a much more common phenomena and that it potentially may be sex specific [15, 20], yet later studies found it challenging to replicate these results both in mouse and human [14, 26]. In our study we identified only 5 genes that clearly show parental imprinting, all of which were previously described as such (*Meg3*, *Zim1*, *Ndn*, *Trapcc9*, *Sgce*). Surprisingly, one gene - *Trappc9*, shows paternal expression in our data, while it is known as a maternally expressed gene in other situations and only one previous report suggested that *Trappc9* gene may be expressed from the paternal allele. Mutations in the maternal allele of *Trappc9* result in mental retardation; therefore, we find it of particular interest that *Trappc9* expression can be switched to paternal allele. Indeed, if that mechanism is inducible, it conceivably may be used to benefit patients with mutations in maternal copy of *Trappc9*.

Another significant aspect of our work is the detailed analysis of ASE calling approach. In this paper, we demonstrate an inclusive approach to ASE analysis, which aims to reduce the amount of filtered SNPs but not at the expense of false positive rate. We show that the artifact introduced by counting closely located SNPs has a significant impact only over a limited distance, which is considerably shorter than the read length. In addition we show that ASE analysis can be reliably performed using the entirety of SNP counts, if using aggregate counts for genes combined with a statistical approach developed for RNAseq. However, it is important to note that F1 data, which effectively does not require phasing of haplotypes, is an ideal test case for our approach.

Application of this methodology to more complex designs, such as human data or other heterogeneous population will require accurate phasing of the haplotypes across each locus. Nevertheless our preliminary comparison of SNP versus gene-based results suggests that this may be a worthwhile effort that can potentially considerably reduce the amount of false positive data. Another possible limitation of our analysis is that summation of SNP counts across the entire locus may mask true signals in cases of isoform specific regulation. To address this we analyzed ASE using the sum of SNP counts over an exon, and tested ASE in an exon by exon manner. We then looked at genes with at least one pair of exons that would show significant ASE but in opposite direction. If isoform specific regulation is indeed a common true biological phenomenon we would expect those genes to have more than one known isoform. Yet, 26 out of 28 cases of discrepant exon pairs occurred in genes with only one known splicing isoform, suggesting that vast majority of these are false positives.

To summarize, our study indicates that along with significant overlap, there are also profound differences between results of *local* and *cis-*eQTL mapping approaches, such that these should be considered complimentary methods of assessing potential *cis*-regulation of gene expression. We believe that our data show that at least some of the previously considered technical artifacts are not sufficient to explain this difference, suggesting that both distant *cis* regulation and strong *trans* regulation play a bigger role in gene expression than previously appreciated. Also, we implement a new approach to analysis of ASE in RNAseq data that does not require SNP filtering on the one hand, but yields fairly conservative results on the other. Using this approach we identified at least >1000 genes that are under *cis* acting regulation in adipose tissue of adult mice, including genes implicated to be involved in adipogenesis and other processes relevant to common human diseases. Finally we identified only a handful of parentally imprinted genes, and find no evidence of genome wide imprinting in our data.

## Methods

### Mice and tissues

All animal experiments were reviewed and approved by the UCLA IACUC. Reciprocal F1 male and female mice were generated by breeding the parental strains in the vivarium at UCLA. F1 pups were weaned at 28 days and fed a chow diet (Ralston-Purina Co) until euthanized at 16 weeks of age, with adipose tissue harvested at that time. Immediately after harvesting, adipose tissue was frozen in liquid nitrogen, and remained frozen at -80°C till RNA extraction. All mice were fed *ad libitum* and maintained on a 12-hour light/dark cycle.

### RNA extraction and QC

RNA was extracted from frozen adipose tissue using Qiazol and Qiagen miRNeasy kit (Cat. #217084) according to protocol, with one modification: to get rid of the lipids we added an additional slow centrifugation step (3 g for 5 min) before doing chloroform extraction. In this step the Qiazol homogenate separates into 3 phases – low, pink, Qiazol with the organic molecules, top – viscous and transparent lipid layer and a thin layer of debris between them. We only used the bottom clean layer to proceed with chloroform extraction and column purification, following the Qiagen protocol exactly. All RNA samples were submitted to BioAnalyzernano-RNA chip analysis, yielding RIN numbers >8.5.

### Library preparation for Illumina sequencing

RNA libraries were made from equimolar pools of RNA, created from 3 BxD and DxB males and females samples. Library preparation was performed as recommended by the manufacturer (Illumina, Hayward, CA,USA). Briefly, PolyA mRNA was isolated and fragmented. First strand cDNA was prepared using random hexamers. Following second strand cDNA synthesis, end repair, addition of a single A base, adaptor ligation, agarose gel isolation of ~200 bp cDNA and PCR amplification of the ~200 bp cDNA. The liver samples were sequenced using the Illumina 1G Genome Analyzer to a coverage of approximately 40 million single end reads of 75 bp. Adipose samples were sequenced with the Illumina HiSeq2000 on paired end 55 bp reads to ~180 million reads per sample.

### Read mapping

In the first step for the ASE detection one has to detect the locations where each read was generated relative to the reference genome, thus we align the reads to the mouse reference genome B (mm9). Given we have pair-end reads of length 50 bp from adipose tissue, we treated each pair as two single-end reads and mapped each one independently. Since paired end reads do not carry independent information, we also repeated the counting process using only one of the mate pairs to be counted. This produced very similar normalized allele specific counts (cor = 0.99) and fold changes (cor = 0.95). We aligned the reads to mm9 genome using mrsFAST [38] allowing up to three mismatches. To help address reference alignment bias, all known D variants were masked to N in the reference genome. This procedure reduced the bias by 2.3 fold, mostly affecting the B specific counts (Additional file 1: Figure S11). The reads were divided into two sets. The first set of reads is the mapped reads, which mapped to the reference genome with three or less mismatches. The second set of reads are those that fail to map to the reference genome. Some of the reads obtained from RNA-seq fail to align to the reference genome as they span the exonic junctions; we refer to this set of reads as unmapped reads. In the next step we map the unmapped reads using the Tophat ( http://tophat.cbcb.umd.edu/ and also see [39, 40]), which is a software design to align the reads to the genome by allowing a break in the reads. Tophat perform the mapping by breaking the read into shorter fragments and map each fragment independently. The reads aligned to the genome in this process were added to the mapped read set. In this step we ignored the reads that map to more than one position in the genome. Thus, only uniquely mapped reads are considered in our analysis. We selected reads with base modifications of the RNA located in one exon, and corresponding to a known genomic SNP between D and B. GEO accession: GSE58239.

### Statistical analysis of ASE

Allele specific expression analysis is discussed in detail in supplementary materials. Briefly, allele specific counts were summed over each gene and strain specific counts were treated as separate RNAseq samples. Differential expression for imprinting or *cis*-eQTL purposes was analysed with DEseq, applying cut-off of FDR corrected p-value < 0.05.

### DNase I hypersensitivity peak analysis

DNase I hypersensitivity peaks data generated by ENCODE project from gonadal fat pads of two male B mice was downloaded through tables tool at UCSC genome browser ( http://genome.ucsc.edu/ENCODE/, wgEncodeUwDnaseGfatC57bl6MAdult8wksPkRep1.narrowPeak.gz and wgEncodeUwDnaseGfatC57bl6MAdult8wksPkRep2.narrowPeak.gz). Narrow peaks, from the two replicates were compared using BEDtools ( http://code.google.com/p/bedtools/), and only peaks that overlap between the two replicates were analyzed. SNPs and indels between B and D were downloaded from Welcome Trust consortium at the Sanger institute ( http://www.sanger.ac.uk/resources/mouse/genomes/), variations were filtered to be polymorphic between D and B strains using in house script.

DNase I hypersensitivity peaks were then overlapped with SNPs and indels using genomic coordinates. For each gene, all peaks within that gene and up to 0-10 kb around the gene were considered. Genes that were assayed for allele specific expression in F1, and had at least 1 peak associated with them, were divided into 3 groups based on adjusted p value (0.05 cutoff) of the ASE and fold change of the alleles – B overexpressed, D overexpressed and neutral. First we calculated the average proportion of peaks harboring a SNP or indel for each group. For quantitative analysis of DNase I peaks, we calculated the proportion of DNase I signal for each gene that is derived from peaks that are not polymorphic in D, we called this proportion an F ratio.

## Electronic supplementary material

Additional file 1: **Includes detailed supplementary materials and methods section.** (DOCX 8 MB)

Additional file 2: **Is a table of all ASE metrics for all of the examined genes.** (XLSX 2 MB)

Additional file 3: **Is a table of information regarding eQTL status in HMDP, F2 or F1 and DNase I hypersensitivity peaks for all genes analyzed.** (XLSX 939 KB)
