# Supplementary material for: Allele-specific expression and eQTL analysis in mouse adipose tissue
Source: BMC Genomics. 2014 Jun 13;15(1):471. doi: 10.1186/1471-2164-15-471 (PMC4089026; doi:10.1186/1471-2164-15-471)
Supplement: Supplementary file 1 — Additional file 1: Includes detailed supplementary materials and methods section. (DOCX 8 MB) [file 12864_2013_6204_MOESM1_ESM.docx]

**Supplementary Information**

**Allele specific expression and eQTL analysis in mouse adipose tissue**

Yehudit Hasin-Brumshtein, Farhad Hormozdiari, Lisa Martin, Atila van Nas, Eleazar Eskin, Aldons J. Lusis and Thomas Drake

**Note: references given refer to the primary manuscript**

***Materials and Methods***

**Allele specific expression analysis**

*Filtering of close SNPs* - one common technical bias in allele specific counts is high concordance of counts between closely located SNPs. This reflects both true underling biological phenomena– correlated transcription of close SNPs, and a technical bias – counts for SNPs that are separated by genomic distances shorter than the sequenced read length, are derived from the same exact reads. While concordant transcription of SNPs is expected and may be exploited to analyze ASE, the technical bias may artificially enhances biological correlation, leading to spurious results. One possible way to resolve this bias is simply to choose only one SNP within the distance equivalent to read length and remove all other from analysis. Yet, if we further consider the effect of this bias it is evident that for reads of length *k*, SNPs that are separated by only 1bp will have a greater number of counts derived from the same reads than SNPs separated by *k-*1 bases. Moreover, with the increasing read length of most sequencing technologies, this correction approach may end up removing significant amount of data from the analysis(~25% of the SNPs for *k* =50bp, ~35% for *k* =100bp reads, Figure S1), undermining the power of ASE analysis while potentially being overly conservative.

Minimum distance between retained SNPs (bp)

Proportion of retained SNPs

Figure S1: Effect of filtering close SNPs on data completeness

Thus, as a first step, we decided to examine the extent of this bias by determining the concordance of total read counts between pairs of consecutive SNPs as function of distance between these SNPs. We focused on pairs of consecutive SNPs (pairs assigned in a sliding window manner, so each SNP may contribute to only two pairs), which reside in the same exon and are separated by 0-100bp. In our case read length is 50bp, therefore reasoning that concordance of counts for pairs separated by 51-100 bp cannot be attributed to using the same reads, and more closely reflects true biological correlation over the same distance as our read length. To our surprise, although our reads are 50bp, SNPs that were separated by more than 18bp did not show greater concordance than SNPs separated by 51-100bp (Figure S1A-D). Thus, for any cluster of SNPs that reside within the same exon or intron and are separated by 18bp or less we only use the first SNP of the cluster in our analysis, and we retained all SNPs that are separated by 19 or more nucleotides.


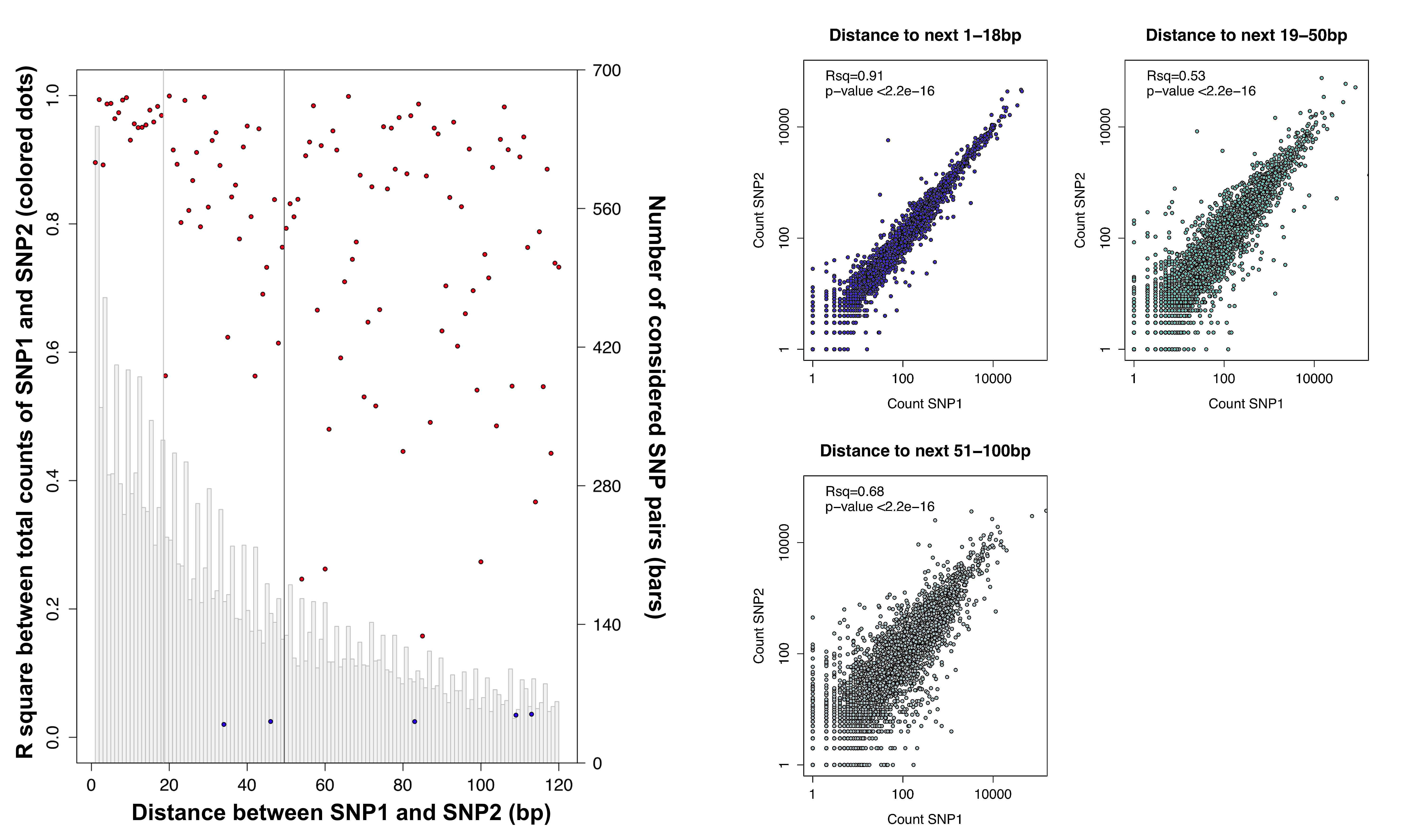


A

B

**Figure S2. Concordance of counts between consecutive SNPs residing in the same exon as a function of distance**.

**A**. R^2^ between the counts of SNP1 and SNP2 (dots, left y axis) as a function of distance and number of examined SNP pairs (bars, right y axis). Grey line was drawn at 18bp (implemented cutoff) and 50 bp (actual read length for this data) **B**. Scatter plots of SNP1 and SNP2 counts in cumulative data over specific distance range (1-18bp, 19-50bp and 51-100bp).

Another technical bias that is inherent to F1 analysis of mouse samples is preferential mapping of B alleles compared to alleles of any other strain (in our case D), as reflected in the average ratio of B to D in all our samples (1.15-1.21, Table 1-*B to D ratio*). This is a known issue in mapping of F1 RNAseq data, typically attributed to using mouse reference genome, which is essentially a B genome. Theoretically, it should be possible to correct for this bias, by constructing a D genome, mapping all data to both references and extracting the best mapping for each read, or alternatively masking each variant base to N – making the effect equal on both strains. Since this would require a considerable bioinformatic effort, we first sought to check whether such approach is likely to alleviate the problem. Allowing up to 3 mismatches in each read, we reasoned that allele specific counts of SNPs which do not have any additional variant within 50bp should not be influenced by this bias and have equal coverage of B and D alleles. Surprisingly, even those SNPs still show a considerable bias towards B mapping (1.09-1.14, Table 1, *B to D ratio* in parenthesis). Therefore, we believe that the simplistic approach of constructing a D reference by replacing the SNPs and using both references for mapping will not be an effective solution, and we chose to proceed with the analysis bearing in mind that we have more power to detect significant overexpression of B alleles than D.

*Choice of statistical test and filtering of low coverage SNPs* - our next goal was to choose a reliable and inclusive statistical approach to determine a list of genes that show significant evidence of ASE. In RNAseq, ASE is measured directly by counting the alleles in reads originated from heterozygous samples. Deriving the ratio of expression in a genome-wide manner typically results an array of values centered around 1, and significant deviation from that ratio is considered evidence of ASE. Yet, since this is a relatively new technique, the statistical approach to determining how much of imbalance is statistically significant evidence of ASE is debatable. The most inclusive approach uses chi-square or Fisher exact test statistic on SNP by SNP basis with some p value cutoff, and defines every gene that has at least one SNP passing the cutoff as significant. Indeed, this approach was used in several earlier studies, but it led to large proportion of irreproducible results (15,21). Subsequently, suggested improvements include read count cutoff for SNPs that are included in the analysis, estimation of false positive rate by considering SNPs in the same exon, and finally aggregation of read counts per gene based on haplotype phasing (14). On the other hand limiting oneself to SNPs with only certain count cutoffs may exclude a considerable proportion of genes from this analysis, and skew the distribution of counts. For example, if we limit ourselves SNPs with 10 reads or more (an arbitrary, but commonly used cutoff) this will reduce the number of assayed SNPs to ~15% and number of genes to ~40% of the original data (Figure S3). Therefore we sought for an approach that will allow us to analyze all SNPs on one hand, but have conservative statistic on the other.

**Figure S3**: Implementing minimal read count cut off drastically reduces the number of examined SNPs (red) and represented genes (black).

Allele specific counts are generated per SNP per sample, and the underlying assumption of the entire ASE analysis is that transcription of both copies of a gene is independent. Under this assumption, allele specific counts can be treated as individual RNAseq samples, using strain as biological condition. Conceptually, this approach is analogous to testing differential expression of genes between different biological conditions, only using a subset of reads that align at polymorphic positions rather than aggregate counts for a genomic region. Testing differential expression of genes is a much more developed application of RNAseq, which received great attention in the recent years. Indeed early papers and methods for RNAseq analysis of genes employed chi-square based statistic, till Anders and Huber and others showed that this approach leads to greater proportion of false positives, and that the negative binomial distribution seem to better represent the underlying noise in distribution of RNAseq read counts (22). Subsequently, in recent years practically all of the most commonly used packages for differential expression of genes, including cuffdiff (http://cufflinks.cbcb.umd.edu/), edgeR (http://www.bioconductor.org/packages/release/bioc/html/edgeR.html) and others utilize this type of statistic. We reasoned that, since SNP specific counts are basically a subset of RNAseq mapped read counts, and likely suffer from the same underlying biases (some of which may be not completely understood), a package for differential expression of genes may be better suited to analyze allele specific counts than a chi-square test. A significant advantage offered by these packages, is that they utilize complete count data without any filtering of the genomic features (SNPs or genes in our case) based on counts or any other criteria, and they were developed to handle samples with different coverage – so they also take into account the mapping bias in our samples. Of the various packages that may be used and which generate comparable results, we chose a relatively conservative one, DEseq, to test for differential expression between alleles and compared the results to use of Fisher exact test on a SNP by SNP basis. To evaluate the utility of DEseq and Fisher exact test we looked at concordance of B to D fold changes of SNPs residing within the same exon, and at number of identified exons with significant ASE (Figure S4). Our assumption was that SNPs in the same exon are transcribed together, thus we expected that for exons with multiple SNPs showing significant ASE the fold change of B to D allele will be favoring the same strain, while discordant fold changes indicate false positive results. We computed both statistics - Fisher exact test and DEseq, for every SNP on a sample specific basis (BxDF, DxBF, BxDM and DxBM). To look at concordance between the samples, for DEseq, we used samples specific B and D counts as replicates in a combined analysis (ALL), while for Fisher exact test we used intersection (ALL) or union (ANY) of all significant SNPs within the four samples. If at least one pair of significant SNPs showed opposite direction of B to D ratio we called this exon discordant. Altogether DEseq had 2-3 fold lower rate of discordant exons (Figure S4A) on sample specific analysis, it identified 60% more significant exons when using all four samples for analysis (969 versus 604, figure S4A “ALL”).

In sample specific tests DEseq is much more conservative than FET, but also much less sensitive. We used two extreme approaches to combining results of FET over multiple samples – “ALL” where only SNPs significant in all samples are deemed as such, or “ANY” which represent the union of all significant SNPs in at least one sample. On the other hand, DESeq employs multiple samples to assess variability within biological condition (strain in our case) and outputs one statistic per SNP “ALL”. Indeed we see that when utilizing all 4 samples, DESeq rate of discordant exons approaches the rate of conservative, while identifying more ASE exons.

**Fig S4: Comparison of DEseq and Fisher exact test for analysis of ASE**

DESeq (red) and Fisher exact test (FET, grey) performance on ASE data. BxDF/BxDM/DxBF/DxBM indicate sample specific analysis. “ALL” indicates analysis that combines all samples (DESeq) or overlapping of significant results from FET. “ANY” indicates union of all significant results from FET. of

**A.** Rate of discordant exons

(% of exons with at least 2 significant SNPs showing opposing effect, out of all exons with multiple SNPs) **B.** Number of identified ASE exons.

We further showed that the number of identified exons with ASE increases proportionally to the number of samples used in our analysis (Figure S5), suggesting that increasing number of samples would considerably expand this list, and that our list of significant ASE is not exhaustive.

**Fig S5: Number of ASE exons is proportional to number of biological replicates.**

Number of ASE exons identified by DEseq depends on variance of allele specific counts, an estimate sensitive to number of available biological replicates.

**A**. Number of ASE exons at 0.05 FDR, using 1-4 samples per condition.

**B.** Relative increase in numbers of identified ASE, as function of number of replicates used.

*Summation of allele specific counts per haplotype*

Another question relevant to the analysis is how to determine genes that have significant ASE from SNP by SNP data. One difficulty is that genes harboring multiple SNPs tend often to have only one or two SNPs being significantly biased, and these may even show discordant allele specific ratio. Another difficulty with SNP by SNP analysis is that while they largely represent the same genes shared between different samples (77% represented in all samples and 83% in at least 3 out of 4) the SNPs themselves are not necessarily the same – only 30% are shared among all samples, and 43% are present in 3 out of.

Aggregating allelic counts over exons or genes is more illustrative of our understanding of the underlying biology but requires accurate phasing of the haplotypes. In this realm, the F1 design offers a significant advantage, since they inherit complete chromosomes from each of the parental strains, therefore allelic counts for each of the strains are phased by definition. We compared ASE analysis of aggregated counts over exons or genes to SNP by SNP approach. First we aggregated SNPs over exons, and analyzed those counts with DEseq using all samples as biological replicates. We then compared this to analyzing exons on SNP by SNP basis (deeming exon as ASE if it has at least 1 significant SNP). The exon level analysis yielded similar numbers in terms of total number of significant exons (1977 versus 2032) with >80% (1660) overlapping between the two. However, the analysis of exon aggregate counts deemed >60% (26 out of 42) discordant exons as not significant.

We further extended this approach to aggregating the allele specific counts over the entire gene region, and looking at concordance of B to D ratio among exons of the same gene. Gene level aggregation reduced the number of genes with significant ASE relative both to exon and SNP based approaches by 30-50%, leaving 1263 genes with significant effect of ASE. In addition, while exon based aggregates yield 39 genes with at least 2 significant exons with opposing B to D fold changes, only 14 of these still showed significant ASE at the gene level. While it is conceivable that discordance between exons within the gene may stem from differential regulation of isoforms, most of the genes (27 out of 39) that have at least two exons with significant ASE and opposite strain preference have only one known isoform in RefSeq, thus probably representing technical limitation rather than true biological phenomenon. Therefore we decided to use aggregate allele specific counts over gene and DEseq for analysis of ASE.

**Supplementary Figures**

**Fig S6: Sex specificity of allele specific expression**

Comparison of ratios of ASE between male and female samples for genes that show significant ASE.

**A**. Ratio of D to B allele in all genes that show significant (FDR adjusted p<0.05) ASE in either sex specific comparisons, or using all samples. Genes with no significant ASE are in grey, genes showing significant ASE in combined analysis are in black, genes showing sex specific significant ASE are in blue (males only) and in red (females only). Values of -4 and 4 were used for 0 and infinity fold changes respectively, for visualization purpose. **B.** Number of genes with significant ASE in sex specific and combined analysis. **C.** Allele specific read counts in the 4 samples for Amyloid beta (A4) precursor Protein-Binding, family A, member 2 gene (Apba2). **D.** Ratio of maternal versus paternal expression of imprinted genes, in male and female samples. Genes on chromosome X show significant parental imbalance only in male samples.


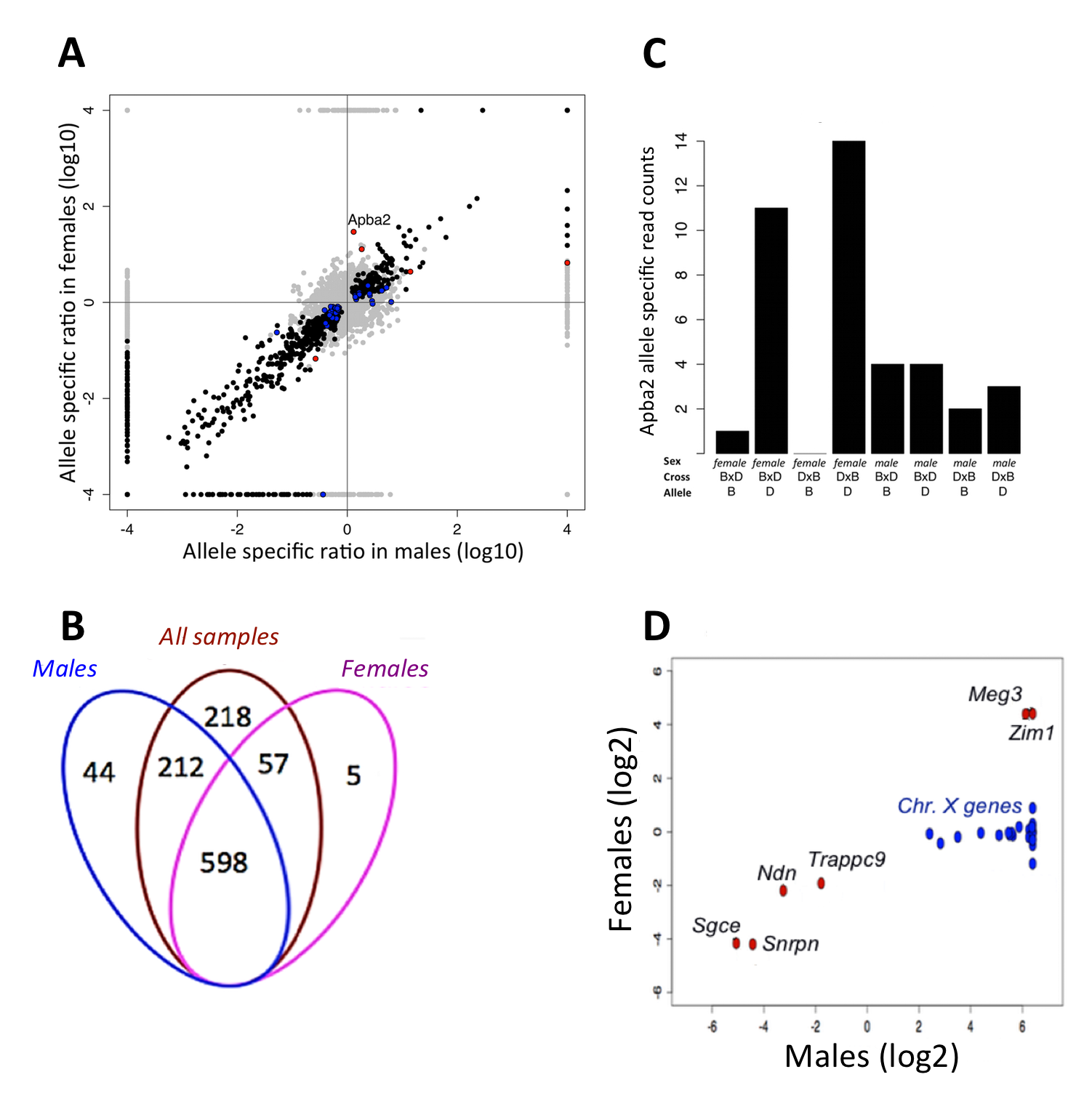


**Fig S7: Expression QTLs in adipose tissue of F2 derived from B and D cross**

**A**. 7622 genes show significant eQTL (LOD>6.2) signal in F2 mice of BxD cross. Each dot indicates the probe position (x axis) and SNP position (y axis). Concentration of signal on the diagonal indicates enrichment for local signals (where the probe and the SNP reside on the same chromosome).
**B**. Some local eQTLs reflect a more distal signal, due to extensive linkage disequilibrium in F2. 1588 genes with local eQTLs (within 2Mb) show a more significant association to another, distal SNP on the same chromosome (“dist”, in blue) while only 111 show a more significant association to a SNP on a different chromosome (“trans”, in red). Size effect of the local association and the distal one is very closely correlated (Rsquare=0.98), while no such correlation is observed for local and trans association.

**
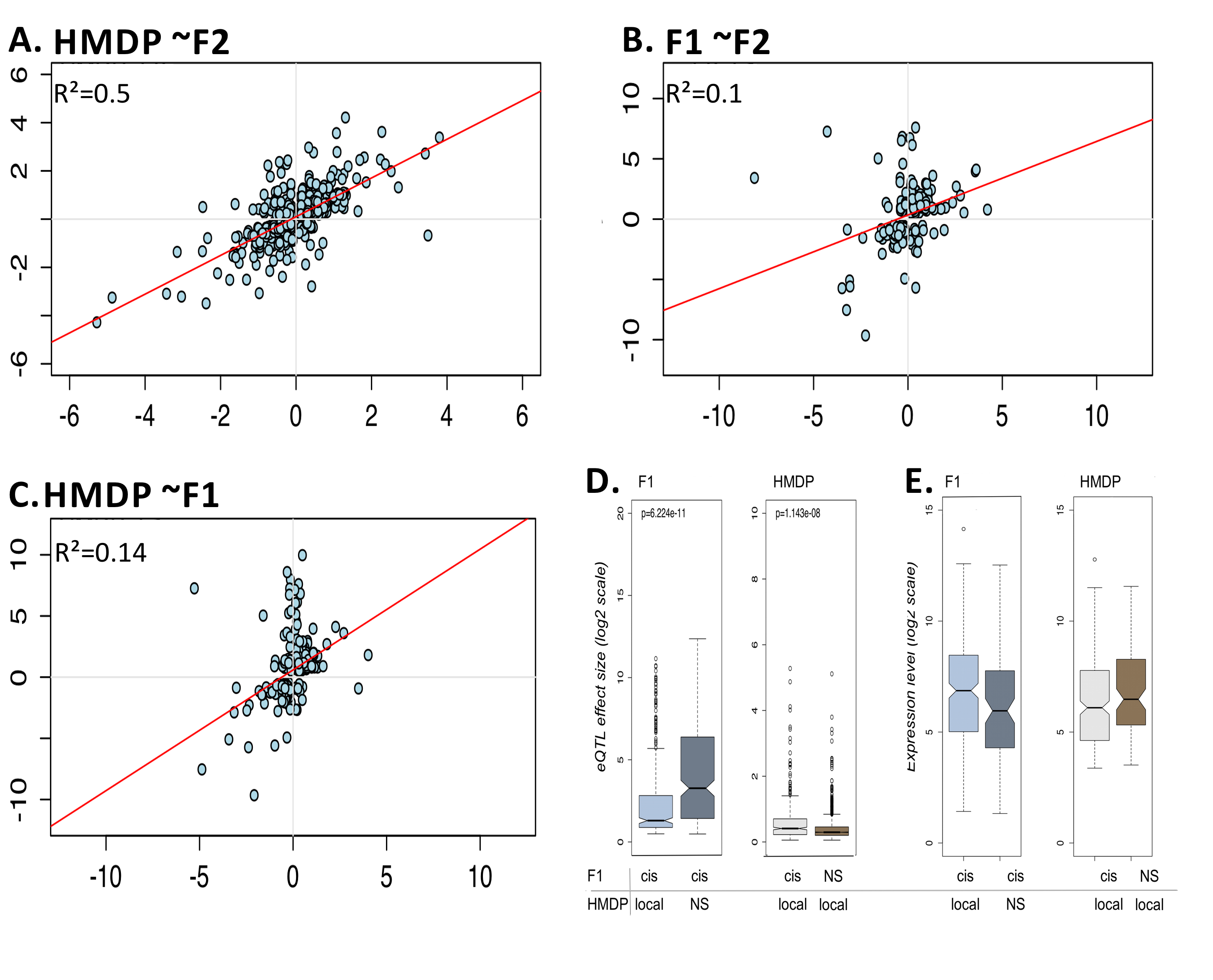
**

**Fig S8: Effect size of local versus cis eQTLs**

Effect size of *local-*eQTLs was determined as ratio of expression between homozygotes for the different alleles, and for *cis*-eQTLs as the fold change between the two alleles.

**A-C**. Comparison of effect sizes between two *local*-eQLT mapping approaches (A), and between *local* and *cis-* eQTLs (B,C). All data are shown on log2 scale, red lines shows linear fit. **D**. Effect size of *cis-*eQTL reproduced by *local*-eQTL are significantly lower (p=6.22e-11) than those of non-reproduced *cis-*eQTLs, while the *local–*eQTL show an opposite trend (p=1.14e-08). **E**. Average expression or counts are not significantly different between *cis*-eQTLs whether they are reproduced or not by *local*-eQTLs.

**Fig S9: Expression characteristic of *cis* eQTLs , grouped by reproducibility in local eQTL studies**

**A.** Distribution of D to B ratio in F1 data. Grey indicates genes with no significant allele specific expression, blue indicates *cis*-eQTLs replicated in both *local*-eQTL studies, yellow are *cis*-eQTLs replicated in HMDP only, green are *cis*-eQTLs replicated in F2 only and *cis*-eQTL detected only in F1 data are in red.

**B**. Normalized total read/number of SNP counts in the different groups. Numbers indicate number of examined gees in each category. Colors are as in A.

**
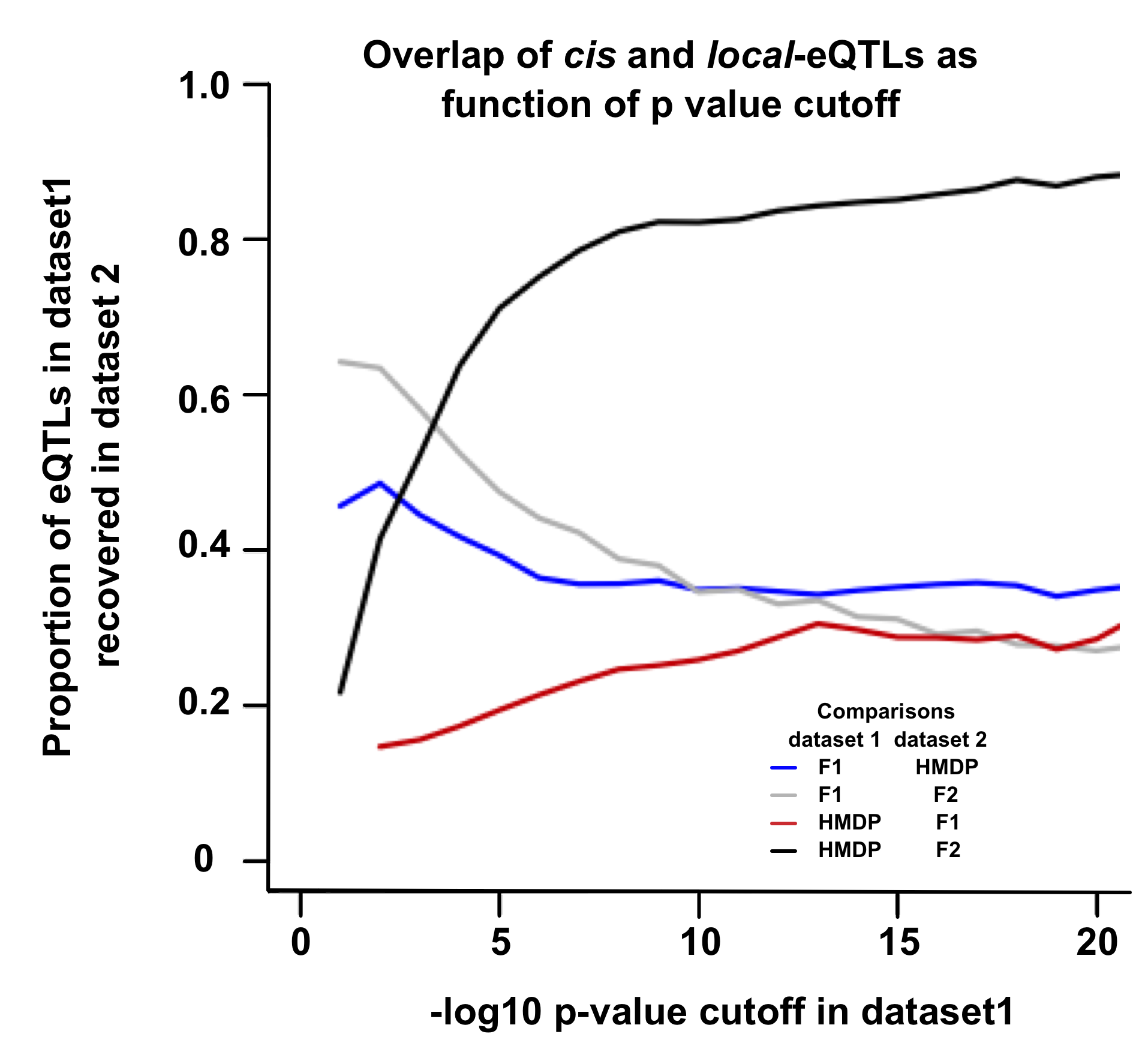
**

**Fig S10: Overlap of *cis* and *local*-eQTLs as function of p-value cutoff**

For each dataset 1 we determined a list of *cis* or *local*-eQTLs that passed the adjusted p-value cutoff indicated on the x axis. We then looked at the proportion of those eQTLS in dataset 2. Significance cutoff for dataset 2 was either adjusted p-value<0.05 (F1 and HMDP) or LOD score >6.2 (F2). Black line indicates comparison between 2 *local*-eQTL approaches, and shows better overlap between datasets at more stringent p-value cutoffs, suggesting that the discrepancy between *local*-eQTL approaches is mostly attributable to lack of power of the F2 cross compared to HMDP. Blue, red and grey lines indicate comparisons between cis and local eQTL approaches, suggesting that the discrepancy is not due to lack of power of the local or cis-eQTLs.

**
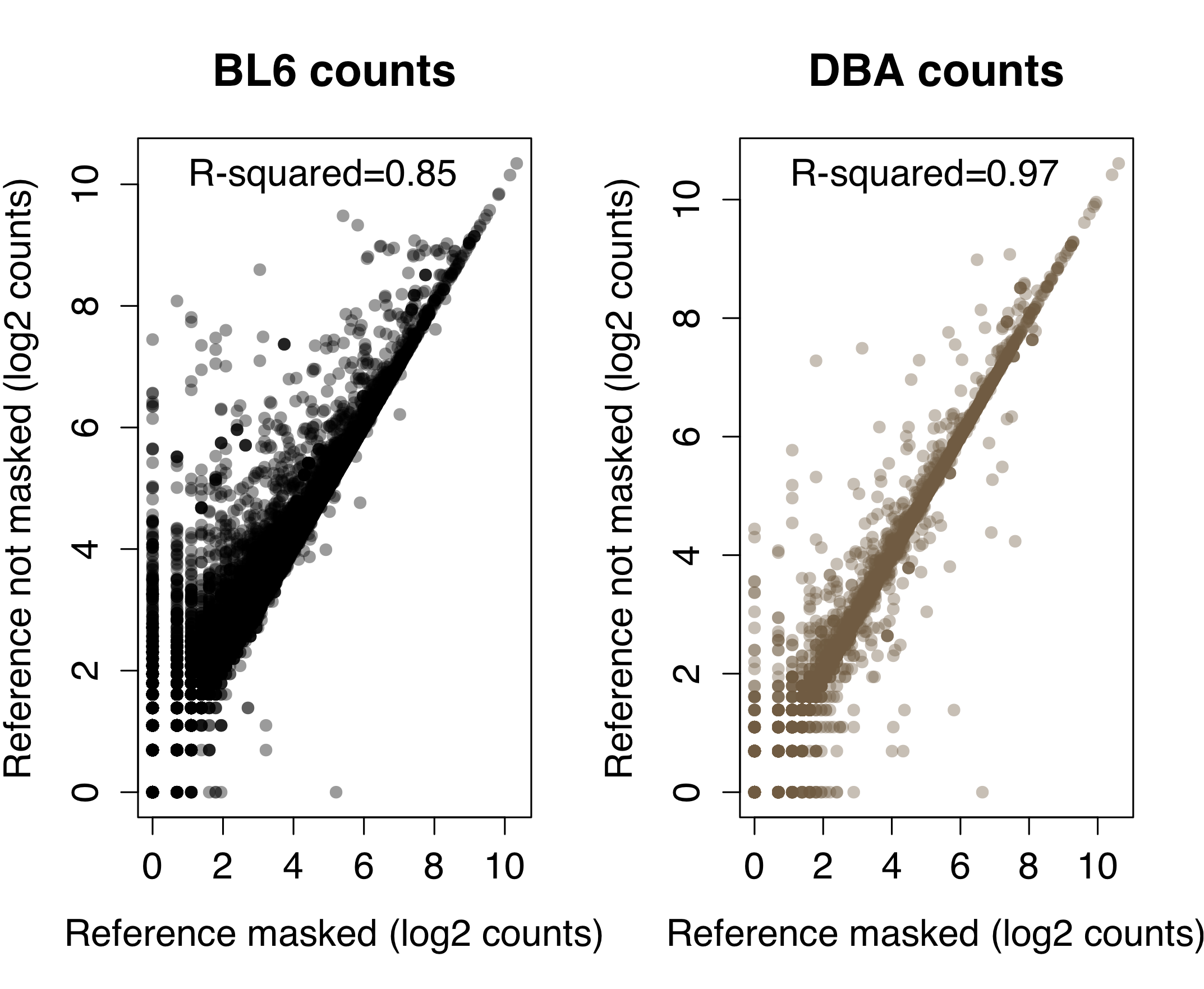
**

**Fig S11: Masking variants in the reference genome reduces the reference alignment bias**

Reads from BxD F1 sample were mapped to a B reference genome, either masking the known D variants to “N” or not. Presented are allele specific counts for B and D alleles, using either of the reference sequences for mapping.

**Supplementary Tables**

Pairwise overlaps of the different datasets with various cutoff conditions. Overlap and recovery rate between the three datasets varies, depending on the selection criteria in the individual datasets. For example all HMDP local eQTLs can be considered, or only the ones associated with a SNP polymorphic in DBA. All possible pairwise comparison are summarized in the table.

**Table S1: Allele specific counts and statistical analysis results by gene**.

Submitted as independent file

**Table S2: Gene based summary of *cis* and *local* eQTL status in different studies.**

Submitted as independent file.

**Table S3: Pairwise comparisons of the different eQTL datasets**

“HMDPall” refers to all significant *local*-eQTLs identified in HMDP, “HMDPdba” refers to all significant local eQTLs identified in HMDP, that are associated with a SNP polymorphic in DBA (“HMDPdba” is a subset of “HMDPall”). “F2_any_Cis” refers to genes that have a significant *local*-eQTL in F2 cross. “F2_max_is_Cis” refers to genes for which the best eQTL association in the genome is significant, and local (“F2_max_is_Cis” is a subset of “F2_any_Cis”). “F1all” refers to genes tat show significant ASE using all 4 samples for ASE analysis, “F1male” refers to genes tat show significant ASE using only male samples for ASE analysis (not a direct subset of “F1all”).

­­­

|  |  | **F1 ASE no exclusive SNPs** | | |
| --- | --- | --- | --- | --- |
| **F1 ASE all exonic SNPs** |  | ***cis*-eQTL** | **No *cis*-eQTL** | **No data** |
|  | ***cis*-eQTL** | 608 | 133 | 344 |
|  | **No *cis*-eQTL** | 16 | 5798 | 1089 |

**Table S4: Exclusion of SNPs with either B or D exclusive expression**

“**F1 ASE no exclusive SNPs** “ indicates analysis module where all SNPs that show an exclusive expression of either B or D alleles were excluded from the original dataset, then analyzed as described in materials and methods. “**F1 ASE all exonic SNPs** “ indicates analysis of all SNPs with filters as described in materials and methods, and used throughout the paper. Numbers indicate number of genes in each category.
